# Open Abdomen and Fluid Instillation in the Septic Abdomen: Results from the IROA Study

**DOI:** 10.1007/s00268-020-05728-3

**Published:** 2020-08-24

**Authors:** Federico Coccolini, Francesca Gubbiotti, Marco Ceresoli, Dario Tartaglia, Paola Fugazzola, Luca Ansaloni, Massimo Sartelli, Yoram Kluger, Andrew Kirkpatrick, Francesco Amico, Fausto Catena, Massimo Chiarugi, the IROA study group, Giulia Montori, Fracensco Salvetti, Ionut Negoi, Monica Zese, Savino Occhionorelli, Sergei Shlyapnikov, Michael Sugrue, Zaza Demetrashvili, Daniele Dondossola, Orestis Ioannidis, Giuseppe Novelli, Mirco Nacoti, Desmond Khor, Kenji Inaba, Demetrios Demetriades, Torsten Kaussen, Asri Che Jusoh, Wagih Ghannam, Boris Sakakushev, Ohad Guetta, Agron Dogjani, Stefano Costa, Sandeep Singh, Dimitrios Damaskos, Arda Isik, Kuo-Ching Yuan, Francesco Trotta, Stefano Rausei, Aleix Martinez-Perez, Giovanni Bellanova, Vinicius Cordeiro Fonseca, Fernando Hernández, Athanasios Marinis, Wellington Fernandes, Martha Quiodettis, Miklosh Bala, Andras Vereczkei, Rafael Curado, Gustavo Pereira Fraga, Bruno M Pereira, Mahir Gachabayov, Guillermo Perez Chagerben, Miguel Leon Arellano, Sefa Ozyazici, Gianluca Costa, Tugan Tezcaner, Matteo Porta, Yousheng Li, Faruk Karateke, Dimitrios Manatakis, Federico Mariani, Federico Lora, Ivan Sahderov, Boyko Atanasov, Sergio Zegarra, Luca Fattori, Rao Ivatury, Jimmy Xiao, Offir Ben-Ishay, Andrea Lippi, Mario Improta, Andrey Zharikov, Vincent Dubuisson

**Affiliations:** 1 General Emergency and Trauma Surgery Department, Pisa University Hospital, Via Paradisia, 1, 56124 Pisa, Italy; 2 General Surgery Department, Ancona Hospital, Ancona, Italy; 3 General and Emergency Surgery Department, Milano-Bicocca University Hospital, Monza, Italy; 4 General, Emergency and Trauma Surgery Department, Bufalini Hospital, Cesena, Italy; 5 General and Emergency Surgery Department, Macerata Hospital, Macerata, Italy; 6 General Surgery Department, Rambam Medical Centre, Tel Aviv, Israel; 7 Department of Surgery, Foothills Medical Centre, Calgary, Canada; 8 Department of Surgery, Trauma Service, John Hunter Hospital, University of Newcastle, Newcastle, Australia; 9 Emergency Surgery Department, Parma University Hospital, Parma, Italy

## Abstract

**Background:**

Open abdomen (OA) is a surgical option that can be used in patients with severe peritonitis. Few evidences exist to recommend the use of intraperitoneal fluid instillation associated with OA in managing septic abdomen.

**Materials and methods:**

A prospective analysis of adult patients enrolled in the International Register of Open Abdomen (trial registration: NCT02382770) was performed.

**Results:**

A total of 387 patients were enrolled in two groups: 84 with peritoneal fluid instillation (FI) and 303 without (NFI). The groups were homogeneous for baseline characteristics. Overall complications were 92.9% in FI and 86.3% in NFI (*p* = 0.106). Complications during OA were 72.6% in FI and 59.9% in NFI (*p* = 0.034). Complications after definitive closure were 70.8% in FI and 61.1% in NFI (*p* = 0.133). Entero-atmospheric fistula was 13.1% in FI and 12% in NFI (*p* = 0.828). Fascial closure was 78.6% in FI and 63.7% in NFI (*p* = 0.02). Analysis of FI in negative pressure wound therapy (NPWT) showed: Overall morbidity in NPWT was 94% and in non-NPWT 91.2% (*p* = 0.622) and morbidity during OA was 68% and 79.4% (*p* = 0.25), respectively. Definitive fascial closure in NPWT was 87.8% and 96.8% in non-NPWT (*p* = 0.173). Overall mortality was 40% in NPWT and 29.4% in non-NPWT (*p* = 0.32) and morality during OA period was 18% and 8.8% (*p* = 0.238), respectively.

**Conclusion:**

We found intraperitoneal fluid instillation during open abdomen in peritonitic patients to increase the complication rate during the open abdomen period, with no impact on mortality, entero-atmospheric fistula rate and opening time. Fascial closure rate is increased by instillation. Fluid instillation is feasible even when associated with nonnegative pressure temporary abdominal closure techniques.

## Introduction

**Fig. 1:**
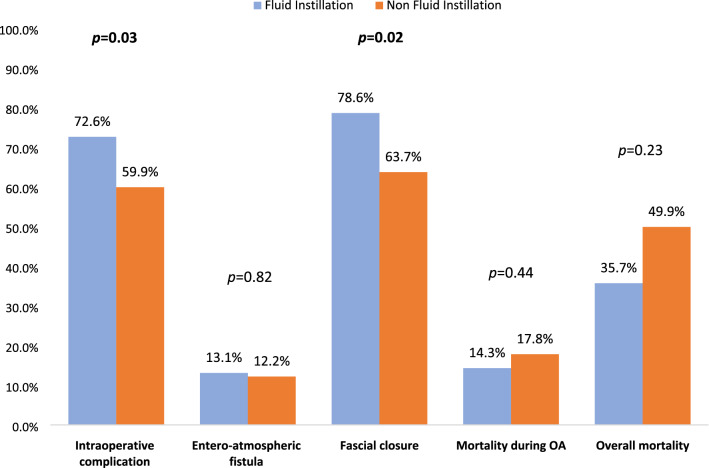
Comparison of fluid instillation between non-NPWT and NPWT groups (*OA* open abdomen)

**Fig. 2:**
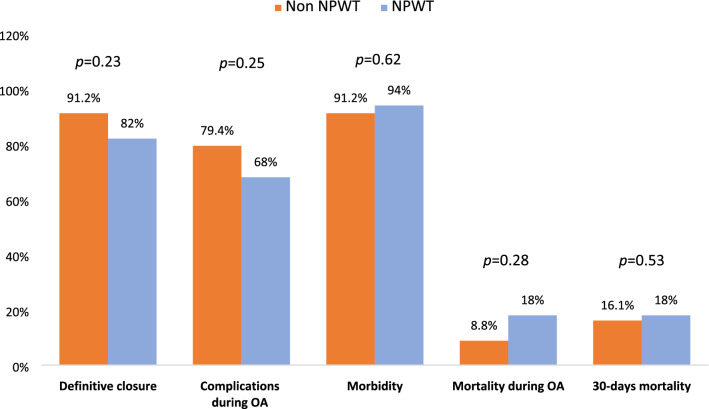
Comparison between fluid instillation and non-fluid instillation groups (*OA* open abdomen)

Abdominal sepsis has an extremely high incidence representing the second most frequent form of sepsis [1]. Treatment of abdominal sepsis and especially of severe sepsis involves the early commencement of correct antimicrobial therapy, adequate hemodynamic support and early source control [2, 3]. To date, despite the progress of medicine, due to the unique anatomical, physiological and microbiological characteristics of the abdominal cavity and viscera, abdominal sepsis remains an extremely serious condition with high mortality rates [4, 5]. Open abdomen (OA) is a surgical option that can be used in patients with severe peritonitis and septic shock. This technique allows the shorter duration of surgery preferred when serious physiological imbalance exists, delaying intestinal anastomoses, to plan a second look when definitive source control is not possible and, prevention of compartment syndrome, if extensive visceral edema exists [6]. However, OA can be associated with serious complications. The definitive closure of the abdomen in patients with peritonitis, compared to those operated for trauma, is often performed after a longer period of time [7–9]. Success rates of delayed fascial closure are lower in patients suffering of peritonitis than in those suffering of trauma, and several studies have identified peritonitis as an independent predictor of fascial closure failure [10–12]. This leads to a greater risk of developing entero-atmospheric fistulas, “frozen abdomen,” intra-abdominal abscesses, a lower rate of definitive fascial closure and a greater risk of developing large abdominal wall hernias [13, 14] (Figs. 1, 2).

Therefore, every effort must be done to obtain early abdominal fascial closure (i.e., as soon as the patient can physiologically tolerate it and as soon as definitive source control is achieved [15]. The different temporary abdominal closure techniques (TACT) present different results in terms of fascial closure rate and entero-atmospheric fistulas (EAF) formation. Several studies have been conducted to evaluate the efficacy and incidence of complications in OA using different TACTs. The World Society of Emergency Surgery (WSES) guidelines provide precise evidence-based guidance on temporary abdominal closure and related treatment, particularly for septic open abdomen patients [6, 14].

In the literature, however, not enough evidence exists regarding intraperitoneal fluid instillation (FI) associated with negative pressure wound therapy (NPWTi) in septic OA [6]. The international register of open abdomen (IROA) is recruiting patients with OA and analyzing results in order to add evidence on the use of OA [16–21].

The present study aims to evaluate the effect of intraperitoneal FI during OA in the treatment of septic abdomen. No intraperitoneal resuscitation has been considered or specifically utilized.

## Materials and methods

IROA is a prospective observational cohort study including patients treated with OA for different diseases. The present study analyzed patients treated with open abdomen for peritonitis. No restriction exists in the type of fluid utilized. Data were collected on a Web platform (Clinical Registers^®^) through a dedicated Web site: www.clinicalregisters.org.

Data were recorded according to the study protocol, approved by the coordinating center Ethical Committee (Papa Giovanni XXIII Hospital, Bergamo, Italy) and registered on ClinicalTrials.gov (ClinicalTrials.gov Identifier: NCT02382770).

The study included both patients with peritonitis present at the time of the first operation and patients with peritonitis complicating a previous surgical intervention.

The only inclusion criterion was patients being treated with OA for peritonitis, and no exclusion criteria were applied. The following details were recorded for all patients according to the study protocol: demographical data, comorbidity, ASA score, indication to the treatment, temporary abdominal closure technique (TACT), duration of OA, length of hospital stay, number of dressing changes, complications, enteric fistula, mortality, abdominal closure, fascial closure and incisional hernia at 1 year. TACTs were summarized in six subgroups (Bogotà bag, Barker vacuum pack, negative pressure wound therapy (NPWT), NPWT with fascial traction, skin closure and Wittmann patch). Moreover, groups were divided into NPWT techniques (Barker vacuum pack, NPWT and NPWT with fascial traction) and non-NPWT (Bogotà bag, skin closure and Wittmann patch) to compare the effects of intraperitoneal fluid instillation (FI) and non-fluid instillation (NFI) between these two subgroups. NPWT systems associated with intraperitoneal fluid instillation were defined as NPWTi.

### Statistical analysis

Continuous variables were expressed as mean and standard deviation and were compared with the ANOVA test; categorical data were expressed as proportions and were compared with the Chi-square test. Linear associations were tested with the Pearson’s linear correlation model. Data about mortality, definitive closure, and number of days with open abdomen were graphically plotted with the Kaplan–Meyer method for the different techniques and indications. (Patients who died during treatment were considered as never closed with a length of treatment = ∞.) All statistical analysis was performed with IBM SPSS 20 (IBM Corp. Released 2011. IBM SPSS Statistics or Windows, version 20.0. Armonk, NY: IBM Corp.).

## Results

The study included 387 patients over the age of 15 years divided into two groups, one consisting of 303 patients, in which peritoneal fluid instillation (NFI) was not applied, and the other one of 84 patients, where this technique was used (FI).

In particular, in 174 patients of the NFI group (57.4%) and in 49 of the FI group (58.3%) the OA was performed during a first surgery for peritonitis, while in 129 (42%) of the NFI group and 35 of the FI group (41.7%) the OA was performed for a peritonitis subsequent to previous surgery (*p* = 0.882).

The two groups were homogeneous for all baseline characteristics excepted for age- and cancer-related comorbidities (Table 1). Most of the patients included in the two groups had an ASA score of III (40.3%) and IV (38%) (Table 1). The different temporary abdominal closure techniques are represented in Table 1.

**Table 1:** Comparison between fluid instillation and non-fluid instillation groups

	Fluid instillation *N* = 84	Non-fluid instillation *N* = 303	All patients *N* = 387	*p*
Age [mean (± SD)]	66.9 (± 14.5)	63 (± 15.3)	63.8 (± 12.2)	**0.03**
Male gender [*n* (%)]	49 (58.3)	147 (48.5)	196 (50.6)	0.11
BMI [mean (± SD)]	27.4 (± 6.2)	27.3 (± 5.0)	27.3 (± 5.2)	0.87
Comorbidities [*n* (%)]				
Cancer and/or chemotherapy	25 (29.8)	130 (42.9)	155 (40.1)	**0.03**
Diabetes	15 (17.9)	44 (14.5)	59 (15.2)	0.45
Immunosuppression/steroids	7 (8.3)	32(10.6)	39 (10.1)	0.54
ASA [*n* (%)]				0.68
I	2 (2.4)	13 (4.3)	15 (3.9)	
II	9 (10.7)	38 (12.5)	47 (12.1)	
III	38 (45.2)	118 (38.9)	156 (40.3)	
IV	32 (38.1)	115 (38)	147 (38)	
V	3 (3.6)	19 (6.3)	22 (5.7)	
Indication [*n* (%)]				0.88
Peritonitis	49 (58.3)	174 (57.4)	223 (57.6)	
Postoperative peritonitis	35 (41.7)	129 (42.6)	164 (42.2)	
Mannheim peritonitis index [mean (± SD)]	23 (9)	23 (10)	23 (± 9)	1

Bold values represent statistically significant results

*BMI* body mass index, *ASA* American Society of Anesthesiology

OA duration, ICU length of stay and overall hospital stay did not differ between the two groups (Table 2). Overall complications were analyzed, both during the period in which the abdomen remained open and after definitive closure. Overall complications were observed in 86.3% of patients in the NFI group and in 92.9% of patients in the FI group (*p* = 0.106). Complications during the OA period were found in 59.9% of patients in the NFI group and in 72.6% of patients in the FI group with a statistically significant difference between the two groups (*p* = 0.034). Complications after definitive OA closure occurred in 61.1% of patients in the NFI group and in 70.8% of patients in the FI group (*p* = 0.133) (Table 2). The analysis of NPWT techniques versus non-NPWT ones showed no statistically significant differences in morbidity and mortality outcomes. Overall morbidity in NPWT techniques was 94% and in non-NPWT was 91.2% (*p* = 0.622) as well morbidity during OA was 68% and 79.4% (*p* = 0.25), respectively (Table 3).

**Table 2:** Comparison between fluid instillation and non-fluid instillation groups

	Fluid instillation *N* = 84	Non-fluid instillation *N* = 303	All patients *N* = 387	*p*
TAC technique [*n* (%)]				**0.04**
Barker vacuum pack	5 (6)	27 (8.9)	32 (8.3)	
Bogotà bag	23 (27.4)	43 (14.2)	66 (17.1)	
NPWT	43 (51.2)	165 (4.5)	208 (3.7)	
NPWT + tension	2 (2.4)	22 (7.3)	24 (6.2)	
Skin-closure	5 (6)	13 (4.3)	18 (4.7)	
Wittmann patch	6 (7.1)	33 (10.9)	39 (10.1)	
OA duration [mean (± SD)]	11 (± 20)	9 (± 19)	9 (± 19)	0.20
Complications during OA [*n* (%)]	61 (72.6)	175 (57.7)	236 (62.8)	**0.03**
Entero-atmospheric fistula [*n* (%)]	11 (13.1)	37 (12.2)	48 (12.4)	0.82
Definitive closure [*n* (%)]	72 (85.7)	249 (82.2)	321 (82.9)	0.44
Fascial closure [*n* (%)]	66 (78.6)	193 (63.7)	259 (66.9)	**0.02**
Overall morbidity [*n* (%)]	78 (92.9)	252 (86.3)	330 (87.7)	0.10
Mortality [*n* (%)]				
During OA	12 (14.3)	54 (17.8)	66 (17.1)	0.44
After definitive closure	14 (16.6)	41 (13.5)	55 (17.1)	0.55
1-year overall	30 (35.7)	130 (42.9)	160 (41.3)	0.23
ICU length of stay [mean (± SD)]	14 (± 12)	17 (± 19)	16 (± 18)	0.17
Total length of stay [mean (± SD)]	16 (± 13)	21 (± 31)	20 (± 29)	0.15
Incisional hernia [*n* (%)]	1 (1.9)	15 (4.9)	16 (9)	0.10

Bold values represent statistically significant results

*NPWT* negative pressure wound therapy, *OA* open abdomen, *ICU* intensive care unit

**Table 3:** Comparison of fluid instillation between non-NPWT and NPWT groups

	NPWT *N* = 50	Non-NPWT *N* = 34	*p*
Age [mean (± SD)]	66 (± 14)	68.53 (± 15)	0.34
Male gender [*n* (%)]	31 (62)	18 (52.9)	0.40
OA duration [mean (± SD)]	14 (25)	6 (3)	**0.03**
Instilled fluid volume per day (liters) [mean (± SD)]	2.7 (1.5)	7 (6.4)	**0.002**
Entero-atmospheric fistula [*n* (%)]	7 (14)	4 (11.8)	0.76
Complications during OA [*n* (%)]	34 (68)	27 (79.4)	0.25
Definitive closure [*n* (%)]	41 (82)	31 (91.2)	0.23
Overall morbidity [*n* (%)]	47 (94)	31 (91.2)	0.62
Mortality [*n* (%)]			
During OA	9 (18)	3 (8.8)	0.28
After definitive closure	9 (22)	5 (16.1)	0.53
1-year overall	20 (40)	10 (29.4)	0.32
ICU length of stay [mean (± SD)]	17 (14)	10 (7)	**0.013**
Total length of stay [mean (± SD)]	19 (15)	9 (6)	**0.027**

Bold values represent statistically significant results

*OA* open abdomen, *ICU* intensive care unit

In NFI group, 37 patients (12%) developed an entero-atmospheric fistula against 11 (13.1%) in the FI group (*p* = 0.828) (Table 2).

Regarding fascial closure, there were missing data in 18.5% of patients in the NFI group and in 14.3% in the FI group. Fascial closure was performed in 63.7% of patients in the NFI group and in 78.6% of patients in the FI group with a statistically significant difference between the two groups (*p* = 0.02) (Table 2). When analyzing definitive fascial closure rate comparing NPWT versus non-NPWT groups, in NPWT the rate was 87.8% vs 96.8% of non-NPWT (*p* = 0.173) (Table 3).

The definitive closure of the abdomen was performed in 82.2% of patients in the NFI group and in 85.7% in the FI group (*p* = 0.446) (Table 2).

Mortality during the period in which the abdomen remained open was 17.8% in the NFI group and 14.3% in the FI group (*p* = 0.446). Mortality rate after abdominal closure was of 16.6% in the NFI group and 13.5% in the FI group (*p* = 0.555). Overall mortality at 1-year follow-up was of 42.9% in the NFI group and 35.7% in the FI group (*p* = 0.236) (Table 2). Overall mortality was 40% in NPWT and 29.4% in non-NPWT (*p* = 0.32) and morality during OA period was 18% and 8.8% (*p* = 0.238), respectively (Table 3).

Incisional hernia rate at 1 year was 10.9% of patients in the NFI group and in 2.5% in the FI group (*p* = 0.103) (Table 2).

## Discussion

The literature has reported up to now only data about NPWTi with no conclusive indications regarding its use. Some authors discourage the instillation of fluids directly in the abdominal and thoracic cavities; others suggest that NPWTi may be useful in reducing OA morbidity and mortality and time of OA and improving fascial closure rates [22]. The need for further evidence to support the use of intraperitoneal FI is standing. Several studies have also published results about peritoneal resuscitation [23–25]. The present study is not analyzing peritoneal resuscitation.

NPWTi has been developed in recent years and consists in the application to the abdominal cavity of a technique originally used for the treatment of infected wounds with the aim of improving the treatment of the abdominal cavity contamination [26]. The rationale of this technique is that instillation may facilitate removal of cellular debris, of exudate and of cytokines and inflammatory mediators. To date, few studies including some case reports showed results about the use of NPWTi with antibiotic-containing solutions, physiological solution or hypochlorous acid in extremely severe abdominal conditions, describing the resolution of the clinical picture without side effects [27, 28]. NPWTi of 0.9% saline solution applied in 48 patients with abdominal sepsis was associated with a reduction in mortality, morbidity rate, ICU and hospital overall length of stay; moreover, it improved fascial closure rate and reduced the incisional hernia rate at 6 months [29]. Sibaja et al. [29, 30] stated that the use of NPWTi can help to promptly control the source of infection, facilitate primary fascial closure and reduce complications rate. Andreano et al. [31] reported that instillation improves local parameters, reduces mortality rates and favors a higher facial closure rate. In 2014, the use of OA NPTWi of 0.9% saline solution in 92 patients treated for abdominal sepsis, compared with 77 patients where only negative pressure was applied, showed an earlier fascial closure and a reduction in hospital length of stay, intraabdominal abscess and EAF rate [32]. In 2019, a prospective study about NPWTi for diverticular perforation was published with no definitive results [33].

Our study analyzes the largest existing cohort, with 387 patients, of whom 84 were treated with FI and 303 without FI. The two groups were homogeneous for all characteristics but for age and the percentage of patients suffering from neoplastic disease, both more represented in the NFI group. Despite being made up of younger and less compromised patients, the FI group registered a greater number of complications, especially during the period in which the abdomen remained open, contrary to what some authors claimed [30, 32]. In the FI group, in fact a higher complication rate was found during the OA period (*p* = 0.034). The overall complications and the complications after abdominal closure were greater in the FI group even if not statistically significant. FI does not seem to have a role in EAF formation. However, considering the small cohort of patients in FI group and the heterogeneity between groups, the small number of events should be considered as a potential risk of bias, as the events/non-events ratio may assume a falsely great value in the FI group. Tao et al. [32] reported different results on EAF. No data exist in the literature regarding EAF and intraperitoneal FI; some authors, however, agreed that FI has an indirect impact on EAF rate by reducing the OA time and improving the infection resolution [30, 32]. The present study did not find significant differences in OA duration linked to FI versus NFI (9 vs. 11 days, respectively), and no statistically significant differences were found in the definitive closure of the abdomen, contrary to what was found in other studies [29, 32]. A statistically significant advantage in fascial closure was furthermore identified in FI group, as shown by previous studies [29–33]. In fact, it should be pointed out that the abdominal closure does not always correspond to fascial closure. To obtain closure of the abdomen together with a complete facial closure would be the optimal result.

The 1-year incisional hernia rate is lower in the FI group. This result is not statistically significant but clinically important and must be kept into consideration because it may be linked to the greater fascial closure rate associated with FI. Contrary to what was shown by other authors, mortality rate is not modified by FI, but the average hospitalization time and ICU admission were shorter in the FI group.

Interestingly, the present study shows how FI may be utilized without negative pressure application using the only natural difference in pressure between the inside and the outside of the abdominal cavity to favor the outflow of fluids. The fact that intraperitoneal fluids are adsorbed by the peritoneal surface with no apparent detrimental effects should also be taken into account.

Data regarding the use of FI in non-NPWT OA techniques are unique, and no previous report exists in the literature on this topic. Patients treated with FI in non-NPWT OA experienced shorter lengths of ICU stay and overall hospital admission: Both results are statistically significant. However, no impact on morbidity was found. The overall 24-h instilled fluid quantity is greater in non-NPWT patients. No difference in complication rate and specifically in EAF rate exists between the two groups. This shows that the effect of FI in increasing the complication rate in the overall population is not determined by the presence or absence of NPWT medication. FI by itself is a favoring factor for complication, even if it has no impact on mortality.

The difference in fascial closure between NPWT and non-NPWT patients may be related to the different OA periods more than the two different techniques. In fact, the non-NPWT group experienced a significantly shorter period of OA.

Data regarding the balance between the instilled fluid volume and the quantity of fluid drained from the peritoneal cavity are lacking. As instilled fluid may be adsorbed by the peritoneum, such data may have helped understanding pathophysiological mechanisms linked to a sort of peritoneal resuscitation during septic OA and its impact on results. Unfortunately, collection of these data is beyond the aim of the present trial.

The present study has some strengths and limitations. Strengths are represented by the large cohort of homogeneous patients enrolled and the completeness of the 1-year follow-up. These patients moreover come from several countries all around the world showing real state-of-the-art OA management with and without FI. Data regarding FI associated with non-NPWT treatment are analyzed and published here for the very first time in the literature. Limitations are the un-distinction within different fluids instilled, the lack of data regarding the balance between instilled and drained fluid volumes and lastly the fact that this is an observational study. Furthermore, the included patients presented abdominal sepsis as a first event or complicating a previous surgical intervention; both NPWT and non-NPWT methods with several techniques for temporary abdominal closure were also considered. This heterogeneity and the multicentric nature of the study may represent both a strength and a limitation of the present study. The observational nature of the present study, however, is due to the impossibility to accrue such large amount of complete data in an emergency surgery setting other that with a design such as a dedicated registry.

## Conclusion

Intraperitoneal fluid instillation during open abdomen in patients with peritonitis seems to increase the complication rate during the open abdomen period, with no impact on mortality, entero-atmospheric fistula rate and open abdomen time. Fascial closure rate seems to be increased by instillation. Fluid instillation is feasible even when associated with non-negative pressure temporary abdominal closure techniques.

## Abbreviations

ASA: American Society of Anesthesiology
OA: Open abdomen
TACT: Temporary abdominal closure techniques
EAF: Entero-atmospheric fistulas
NPWT: Negative pressure wound therapy
FI: Fluid instillation
NFI: Not fluid instillation
NPWTi: Intraperitoneal fluid instillation associated with negative pressure wound therapy
